# Identification of maternally-loaded RNA transcripts in unfertilized eggs of *Tribolium castaneum*

**DOI:** 10.1186/1471-2164-13-671

**Published:** 2012-11-27

**Authors:** Kevin M Preuss, Jacqueline A Lopez, John K Colbourne, Michael J Wade

**Affiliations:** 1 Department of Biology, Indiana University, Bloomington, IN, USA; 2 Center for Genomics and Bioinformatics, Indiana University, Bloomington, IN, USA; 3 Present Address: Department of Biology, Drury University, Springfield, MO, USA

**Keywords:** Maternal transcriptome, Maternal effects, Sexual antagonism, Whole-genome microarray

## Abstract

**Background:**

Maternal RNAs play a critical role in early development. Variation in the diversity and levels of maternally derived gene transcripts may be central to the origin of phenotypic novelty -- a longstanding problem in evolution and development. By studying maternal transcriptomes within and between divergent species, a better understanding of the evolutionary forces acting on maternal RNA allocation is possible.

**Results:**

We present the first maternal transcriptome of the red flour beetle, *Tribolium castaneum*. Using a tiled whole-genome microarray, we found that 58.2% of *T. castaneum* genes are maternally loaded into eggs. Comparison of known *Drosophila melanogaster* maternal genes to our results showed widespread conservation of maternal expression with *T. castaneum*. Additionally, we found that many genes previously reported as having sex or tissue specific expression in *T. castaneum* were also maternally loaded. Identification of such pleiotropy is vital for proper modeling and testing of evolutionary theory using empirical data. The microarray design also allowed the detection of 2315 and 4060 novel transcriptionally active regions greater in length than 100 bp in unfertilized and fertilized *T. castaneum* eggs, respectively. These transcriptionally active regions represent novel exons of potentially unknown genes for future study.

**Conclusions:**

Our results lay a foundation for utilizing *T. castaneum* as a model for understanding the role of maternal genes in evolution.

## Background

Maternal resources are essential to initiate development and to embryo growth. Early embryonic development in animals is a collaborative effort whose control is shared by genes in both the maternal and embryonic genomes. Thousands of RNA transcripts and proteins from the maternal genome are deposited into the egg during oogenesis. These maternal gene transcripts direct and sustain embryonic development until they are replaced with zygotic transcripts [1,2]. Maternal RNAs are known to be important for regulating cellular and developmental processes as well as establishing embryonic axes [3-6]. Evolutionary differences in the maternal RNAs may thus affect the future developmental trajectory of an embryo.

Because of their critical influence on early development, maternal RNAs are believed to play an important role in the developmental differences among species [5,7]. Thus, identifying maternal RNA transcripts is an important step for studying differences in development between taxa. We use recent technological advances in DNA tiling arrays to identify maternal transcripts deposited in the oocyte prior to fertilization in the red flour beetle, *Tribolium castaneum*, characterizing the eggs laid by virgin female beetles. We compare these transcripts to those present in 24-hour fertilized eggs to identify the array of early acting genes after fertilization. The identification of maternal transcripts introduced here will spark future work to identify species-specific and conserved maternally acting genes.

Because maternal genes are transmitted via RNA across generations, they are uniquely situated to position offspring to meet environmental challenges. In some species, the composition of oocyte maternal transcripts is believed to be influenced by the environment, resulting in offspring polyphenisms [8-10] which in turn affect the evolutionary and selective forces acting on genes. Thus, identifying maternally contributed RNAs and studying their regulation may yield insights into how trans-generational processes affect the development of complex phenotypes.

Evolutionary theory [7,11] has shown that genes with strictly maternal expression evolve differently than constitutively expressed genes, because selection on sex-specific genes is weaker [11-14]. However, the strength of selection on genes with *both* maternal and zygotic function may be equal to or greater than that of a similar gene with only zygotic function [7,11,15]. Thus, the ability to accurately assign genes with pleiotropic function to a specific expression pattern allows more accurate tests of theoretical predictions with empirical data.

To characterize the maternal transcriptome, we identified RNAs present in unfertilized eggs and in early developing eggs (≤24 h post laying) of *T. castaneum*, an emerging model system. Additionally, our design also allowed us to identify novel transcriptionally active regions (TARs) outside of the annotated genes. These TARs represent potential transcripts not yet included in the *T. castaneum* genome as it is currently annotated. These novel results provide a framework for utilizing *T. castaneum* as a model for understanding the role of maternal genes in evolution.

## Results

### Maternal expression in virgin eggs using a tiled whole-genome microarray

We used a tiled whole-genome microarray to identify transcripts present in total RNA isolated from unfertilized and fertilized 24 hour egg collections at 30°C. The microarray design allowed us to test expression levels for 14,323 (99.1%) of the 14,460 annotated genes on the assigned chromosomal linkage groups of the Tcas_3.0 assembly [16]. We found that 58.2% (8,333) of the testable annotated genes on assigned chromosomes (14,323) were expressed in unfertilized eggs (for individual gene results, see Additional file 1). All of the maternally expressed genes in the unfertilized eggs were also present in the fertilized eggs. Fertilized eggs (≤24 hours post-laying), which contain both maternal and zygotic transcripts, express only 893 more annotated genes than the maternally loaded complement found in unfertilized eggs. Our expression results agreed with the previously reported expression of known early developmental genes in *T. castaneum* (see Additional file 2: Table S1). We found a total of 8,158 genes differentially expressed between unfertilized and fertilized eggs (q-value < 0.1). Of those 8,158 genes, 779 (9.6%) had twofold or greater expression in unfertilized eggs than in fertilized eggs. Approximately twice that number of genes, 1,749 (21.4%), had a twofold or greater expression level in fertilized eggs relative to unfertilized eggs.

**Figure 1:**
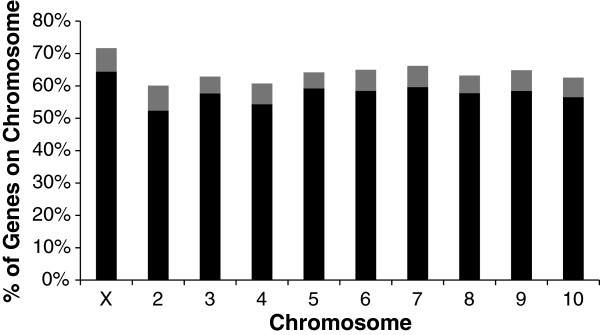
**Expressed Genes by Chromosome.** The percentage of total annotated genes on the indicated chromosome that are expressed in 0–24 hour eggs are depicted with the total height of the bar on the graph. The percentages of genes which are maternally loaded into eggs are depicted in black while those additional genes that are only expressed in fertilized eggs are depicted in grey.

We examined the chromosomal location of maternally expressed genes in the unfertilized eggs (Figure 1). We found that the X-chromosome (64.8%) was enriched with maternally loaded annotated genes, relative to the autosomes (autosomal mean = 57.8%; G-test of heterogeneity among chromosomes: G = 46.41; df = 9; p << 0.01). There are more maternally loaded genes on the X-chromosome than expected based on gene number alone.

### Expression of maternal *Drosophila melanogaster* orthologs

**Figure 2:**
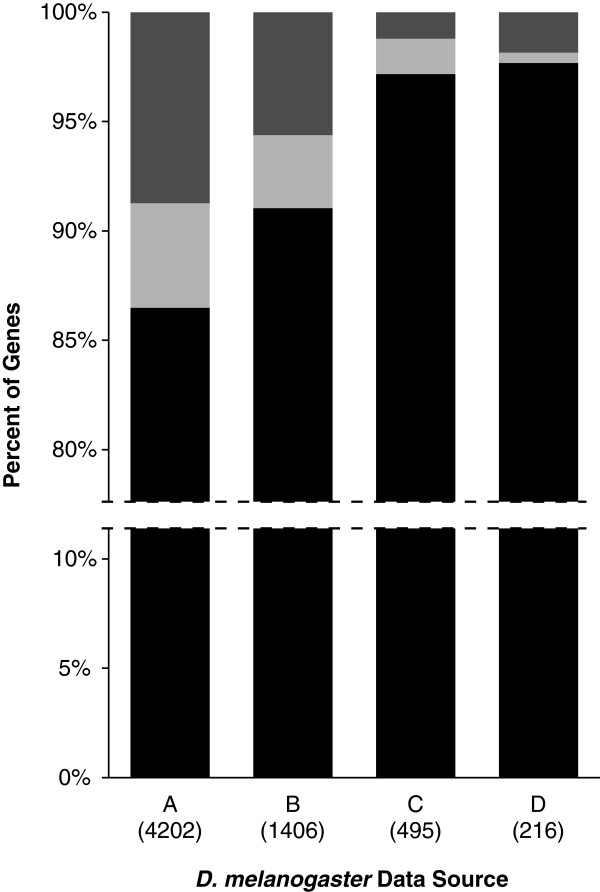
**Expression of *D. melanogaster *maternal gene orthologs.** The percentages of previously identified *D. melanogaster* maternal genes which have detectable *T. castaneum* orthologs that are maternally loaded (Group A, black bar), only zygotically expressed (Group B, light grey bar), or not expressed (Group C, dark grey) in 0–24 hour eggs. Numbers of genes from sources are indicated in parentheses. *Drosophila melanogaster* data sources: (**A**) Berkeley *Drosophila* Genome Project in situ database [24,25], (**B**) Fly-Fish database [26], (**C**) Renzis, *et al*. [34], (**D**) Arbeitman, *et al*. [23].

We examined the expression of *T. castaneum* orthologs of known maternal genes in *Drosophila melanogaster* from four publically available datasets (Figure 2). Among the datasets, 86.5% to 97.7% of the *D. melanogaster* maternal genes with a *T. castaneum* ortholog on assembled chromosomal scaffolds had at least one *T. castaneum* ortholog that was maternally loaded into *T. castaneum* eggs (Group A). Between 0.5% and 4.8% of the *D. melanogaster* maternal genes had *T. castaneum* orthologs which were zygotically expressed in fertilized eggs, but not maternally expressed in unfertilized eggs (Group B). There were 1.2% to 8.7% of the *D. melanogaster* maternal genes with orthologs that were not expressed in 0–24 h *T. castaneum* eggs (Group C). We also analyzed the gene ontology categories of the three groupings of *D. melanogaster* genes for enriched terms. The four enriched terms with the smallest p-value of each grouping using the Berkeley *Drosophila* Genome Project *in situ* database comparison are displayed in Table 1 (complete results available in Additional file 3). Of the 4202 *D. melanogaster* genes from the Berkeley *Drosophila* Genome Project *in situ* database with *T. castaneum* orthologs, 3718 (88.5%) of the *D. melanogaster* genes had associated gene ontologies. Of the 3718 *D. melanogaster* genes with associated gene ontologies, 3235 (87.0%) were in group A, 184 (4.9%) were in group B, and 299 (8.0%) were in group C.

**Table 1:** **Enriched molecular functions of compared *Drosophila melanogaster *maternal genes**

**Group**	**GO term**	**P**-**value**	**Sample frequency**	**Background frequency**
A	small molecule binding (GO:0036094)	5.17e-08	525/3235 (16.2%)	552/3718 (14.8%)
	nucleoside phosphate binding (GO:1901265)	1.24e-07	486/3235 (15.0%)	510/3718 (13.7%)
	nucleotide binding (GO:0000166)	1.24e-07	486/3235 (15.0%)	510/3718 (13.7%)
	organic cyclic compound binding (GO:0097159)	1.24e-07	486/3235 (15.0%)	510/3718 (13.7%)
B	sequence-specific DNA binding transcription factor activity (GO:0003700)	5.44e-04	35/184 (19.0%)	290/3718 (7.8%)
	nucleic acid binding transcription factor activity (GO:0001071)	5.44e-04	35/184 (19.0%)	290/3718 (7.8%)
	structural constituent of chitin-based cuticle (GO:0005214)	4.36e-03	11/184 (6.0%)	41/3718 (1.1%)
	structural constituent of cuticle (GO:0042302)	7.28e-03	11/184 (6.0%)	43/3718 (1.2%)
C	endopeptidase activity (GO:0004175)	6.26e-10	37/299 (12.4%)	124/3718 (3.3%)
	serine-type endopeptidase activity (GO:0004252)	5.99e-09	21/299 (7.0%)	45/3718 (1.2%)
	peptidase activity, acting on L-amino acid peptides (GO:0070011)	8.20e-08	41/299 (13.7%)	171/3718 (4.6%)
	serine-type peptidase activity (GO:0008236)	4.22e-07	21/299 (7.0%)	54/3718 (1.5%)

The four enriched molecular function terms with the lowest p-values for each group within the comparison of the Berkeley *Drosophila* Genome Project in situ database maternal genes to the *T. castaneum* findings. Group A consists of *D. melanogaster* maternal genes with *T castaneum* maternally loaded orthologs. Group B consists of *D. melanogaster* maternal genes with *T. castaneum* orthologs which were expressed in fertilized eggs but not maternally loaded. Group C consists of *Drosophila melanogaster* maternal genes with *T. castaneum* orthologs which were not expressed in eggs.

### Comparison of maternal expression and female-biased expression genes in *T. castaneum*

Overall, 1254 (75.5%) of the 1661 testable female expression-biased genes identified in *T. castaneum* by Prince, *et al*. [17] were present as maternal transcripts in unfertilized eggs; this substantially exceeds the expected frequency based on the overall average of 58.2% of genes expressed in unfertilized eggs as maternal transcripts (χ^2 ^= 204.266, df = 1, p < 0.01). Similarly, the 1260 transcripts of autosomal genes with female-biased expression in the study of Prince, *et al*. [17] were enriched in unfertilized eggs (989 of 1260 female expression-biased autosomal genes, 78.5%) relative to the overall number of total autosomal genes with expression in unfertilized eggs (7819 of 13530 autosomal genes, 57.8%) (χ^2^ = 221.176, df = 1, p < 0.01). However, for the 401 X-linked female-biased expression genes, 265 (66.1%) were also found as transcripts in unfertilized eggs. This finding is not different from the expectation based on the number of total X-linked genes (514 of 793 X-linked genes, 64.8%) (χ^2^ = 0.290, df = 1, p > 0.5). Thus, autosomal genes with adult female-biased expression are enriched for maternal expression in unfertilized eggs, but similarly female-biased expressed genes on the X-chromosome are not.

### Comparison of maternal expression and male-linked genes in *T. castaneum*

**Figure 3:**
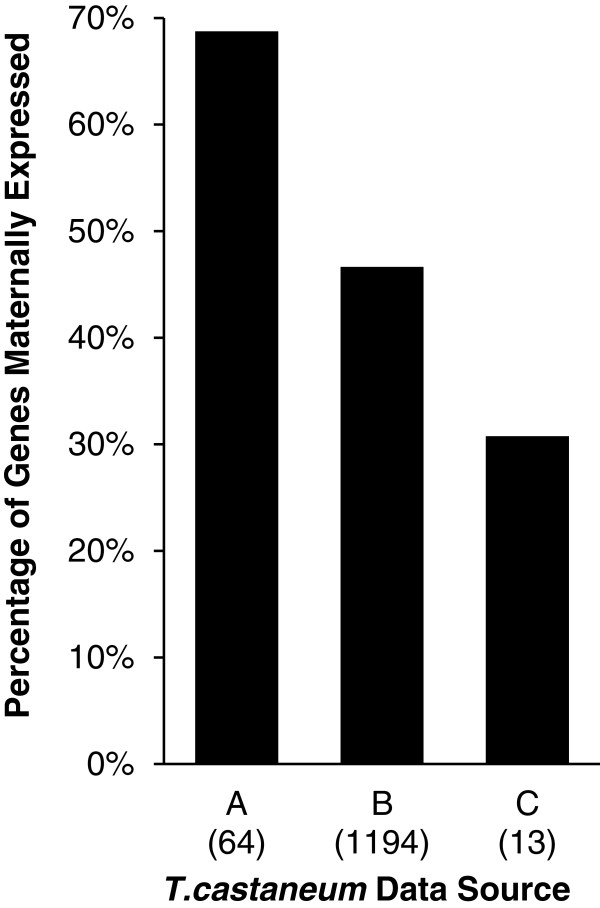
**Pleiotropic male-linked genes.** The percentage of male-linked genes identified in previous publications that are maternally expressed in 0–24 hour unfertilized eggs. Numbers of genes from sources are indicated in parentheses. Data sources: (**A**) Parthasarathy, *et al*. [18], (**B**) Prince, *et al*. [17], (**C**) South, *et al*. [19].

Genes in *T. castaneum* have been linked to male function by their expression in male accessory glands [18], by patterns of male-biased whole-body gene expression [17], or mass spectrometry of seminal fluid proteins [19]. We compared these genes designated male-biased in these earlier studies to the genes whose maternal transcripts were found in unfertilized eggs. Surprisingly, we found that virgin females load RNA transcripts into unfertilized eggs for 68.8% of male accessory gland genes, 46.6% of male-biased whole-body genes, and 30.8% seminal fluid protein genes (Figure 3).

### Novel transcriptionally active regions

Using a previously published methodology [20], our analysis also revealed the presence of novel “transcriptionally active regions” (TARs). These are defined as contiguous regions of expression that do not overlap with annotated genes within the genome. Intron size in the *T. castaneum* genome is non-normally distributed, with a primary peak at 57 bp and a secondary peak at 1200 bp, while exon size has a unimodal average of 310 bp [21]. We examined only TARs longer than 100 bp (greater than the average length of two overlapping sequential probes) located more than 100 bp (larger than most introns but smaller than average exon size) away from the nearest annotated gene on assigned chromosomal contigs. We found 2315 and 4060 TARs in unfertilized and fertilized eggs, respectively. There were 485 TARs unique to unfertilized eggs, with no overlap with TARs from the fertilized eggs. In fertilized eggs, there were 2304 TARs that were unique, with no overlap with TARs from unfertilized eggs.

**Figure 4:**
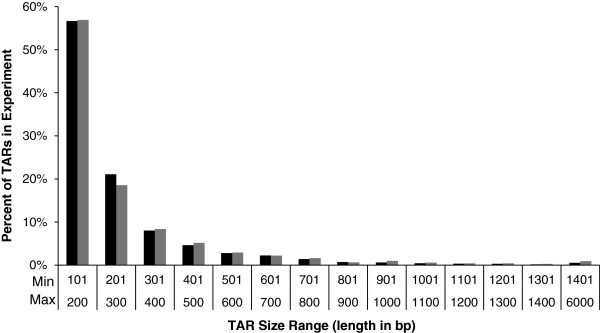
**Size distribution of Transcriptionally Active Regions (TARs) >100 bp.** The percentage of total TARs within a given size range expressed in 0–24 hour eggs are depicted with the height of the bar on the graph. The percentages of TARs identified from the unfertilized egg collection are depicted in black while the percentages of TARs identified in fertilized eggs are depicted in grey.

These TARs represent novel exons of potentially novel genes. The size distributions of the TARs from unfertilized and fertilized eggs were not different (Figure 4; Kolmogorov–Smirnov test, p=0.9971, k = 0.1429). The number of TARs per chromosome was very highly correlated with chromosomal length in both unfertilized and fertilized eggs (correlation coefficients = 0.919, 0.943 respectively).

## Discussion

Our study is the first unbiased, genome-wide survey of maternally-loaded transcripts in unfertilized eggs of *T. castaneum*. We find the majority of annotated genes (58.2%) are maternally loaded as transcripts into eggs prior to fertilization. Our findings reinforce previous work indicating that a large fraction of genes are maternally loaded into eggs [1,22,23]. Our findings provide a foundation for studying the evolutionary dynamics of trans-generational processes.

### Early expression dynamics

Early development in metazoans is a collaborative effort between the maternal RNAs allocated to the egg and the expression of early zygotic transcripts. Maternal RNAs are often rapidly degraded as part of the developmental process [1]. We identified 779 maternal genes with a more than two fold greater amount of RNA in unfertilized eggs relative to fertilized eggs. Other groups have taken such changes in expression level as indicative of maternal RNAs which are degraded as part of the developmental process [23-26]. Additionally, we found 1,749 genes which were showed a greater than two fold increase in expression level in fertilized eggs relative to unfertilized eggs, suggesting that these genes are early expressed zygotic genes. The proper regulation of RNA transcripts within the developing zygote is vital to ontogeny. Both of these gene groups represent potentially important candidate genes in studying the maternal-to-zygotic transition in *T. castaneum*.

### Conservation of maternal gene expression in insects

Because of their critical role in early development, maternal genes may play an important role in producing phenotypic differences between species. We compared four previously published datasets identifying maternal genes from *Drosophila melanogaster* to our findings from *T. castaneum*. We found that 86.5% to 97.7% of *D. melanogaster* maternal genes had a *T. castaneum* ortholog that was also maternally deposited into eggs (Group A). Our analysis of the gene ontology functions of this group showed an enrichment of genes related to maintaining basic cellular and metabolic processes. The common maternal transcriptome of these two species thus contains genes which are fundamentally important for the basic cellular functions of the developing embryo.

We also found that 0.5% to 4.8% of *D. melanogaster* maternal genes had a *T. castaneum* ortholog that was zygotically expressed in early fertilized *T. castaneum* eggs, but was not maternally loaded into the eggs (Group B). Although these genes are expressed during early embryonic development in both *T. castaneum* and *D. melanogaster*, they have either acquired maternal gene expression in *D. melanogaster* or lost maternal gene expression in *T. castaneum*. These genes were enriched for gene ontology terms related to transcription factor activity and cuticle structural elements. It is possible that these genes function similarly in the ontogeny of both species despite the divergence in expression. If so, these genes may be of interest for studying the evolutionary forces affecting the maintenance or acquisition of maternal gene expression. Alternatively, the difference in expression between these two species may be related to morphological and developmental differences between these species. Under these conditions, the acquisition or loss of maternal gene expression may be involved in novel trait development. Understanding the evolutionary forces that have altered the expression of these genes in each species may yield insight about the forces affecting either the origination or elimination of maternal gene expression and possibly the origination of morphological novelties.

Finally, a few *D. melanogaster* maternal genes (1.2% to 8.7% depending on *D. melanogaster* data source) had no detectable expression of their *T. castaneum* orthologs in either fertilized or unfertilized eggs, indicating that these genes may be important for early embryonic development in *D. melanogaster*, but not *T. castaneum* (Group C). This group of genes was enriched primarily for different forms of peptidase activity. These findings suggest that dietary differences between *T. castaneum* and *D. melanogaster* may play an important role in species-specific gene expression. Although the amino acid requirements of *Tribolium* and *Drosophila* species may be similar [27], each species may be adapted to a particular protein source. Further study of such species-specific genes may give insight into the unique aspects of *D. melanogaster* development.

Although our findings warrant further investigation, such basic groupings should assist in the identification of candidate genes involved in general early insect development as well as those genes that may be involved in the specific developmental differences between *T. castaneum* and *D. melanogastser*. These types of comparisons make it possible to target general versus species-specific developmental processes, thus providing as starting point for investigation into the evolutionary dynamics affecting maternally expressed genes.

### Genomic location of maternally loaded and female-biased expression genes

The X-chromosome of *T. castaneum* is known to be enriched for female expression-biased genes [17]. Genes that are maternally loaded into eggs must be transcribed at levels sufficient for egg production and may therefore be more highly expressed in females. Thus, the location of maternally loaded genes within the genome may influence the location of female-biased expression genes. Indeed, we found that maternally loaded genes were also enriched on the X chromosome of *T. castaneum* (Figure 1). On the autosomes, we found that female-biased expression genes were more likely than expected to also be maternally loaded. However, on the X chromosome, female-biased expression genes are not enriched for maternal gene function. These findings suggest that although the basis for the female-bias expression for autosomal genes may be related to maternal loading into eggs, this is not the case for female-biased expression genes located on the X chromosome. These findings suggest the feminization of the X chromosome observed by Prince, *et al*. [17] is unlikely due to the location of maternal genes. Furthermore, although most of the female-biased expression genes in *T. castaneum* are also maternally loaded genes, the locations of genes which are both female expression-biased and maternally loaded are skewed against the X-chromosome and in favor of an autosomal location. Thus, the forces affecting gene movement, such as sexually antagonistic selection, may be acting differently on genes that are only female-biased or maternally loaded than those genes that are both female expression-biased and maternally loaded.

### Pleiotropic gene functions

In any model system, particularly in emerging systems like *T. castaneum*, it is important to understand genes in the context of the organism as a whole. Recent studies in *T. castaneum* have examined specific evolutionary or developmental aspects of genes, classifying them as sex or tissue specific. However, we find that many of genes with reported male-linked function are also found in our unfertilized egg samples (Figure 3). This indicates that sex-biases in gene expression in adult tissues may not be sufficient to classify a gene as male or female specific in its function(s). Such widespread pleiotropy must be taken into account in the modeling and testing of population genetic theory of sexual selection processes. Indeed, our data on maternally loaded genes in *T. castaneum* should not be construed as having strict-maternal expression. Although our data can indicate a gene is maternally expressed and deposited into eggs, we cannot exclude later zygotic expression. Taken together, these results indicate that more gene expression data from different life-stages and sexes is needed to classify sex or tissue specificity.

### Prevalence of TARs

One of the advantages of the microarray design in the present study is the ability to identify throughout the genome transcriptionally active regions (TARs) not associated with annotated genes. These TARs represent the exons of genes, which include both potentially novel protein coding and non-coding genes. We found 2315 and 4060 TARs longer than 100 bp in unfertilized and fertilized eggs, respectively, representing over 526 and 922 potentially novel genes assuming the average number of exons per gene of 4.4 [21]. We also found 485 TARs which were unique to unfertilized eggs. These 485 TARs may represent yet unknown maternal transcripts which are rapidly degraded upon fertilization. We found that the distribution of TAR locations was highly correlated to chromosomal length, which is similar to the distribution of annotated genes. This suggests that TARs may represent types of exons or genes which, although widespread, are not typically detected by the current annotation methods. Further investigation of these TARs will provide a clearer picture of the diversity of gene types and features which may improve the annotation process, which is particularly important given the number of novel genomes being sequenced.

## Conclusions

Our study provides the first transcriptome of maternally loaded RNAs of a representative species of the largest eukaryotic order. These findings have allowed for the identification of common and species-specific maternally loaded genes between *D. melanogaster* and *T. castaneum*, making it possible to begin to explore the divergence among taxa of early developmental processes. The high prevalence of maternally loaded genes in *T. castaneum* also highlights the necessity for understanding the many contexts in which a gene functions in order to model and test population genetic theory. We also detected a large number TARs, representing the discovery of many potentially novel genes. These results provide a framework for utilizing *T. castaneum* as a model for understanding the role of maternal genes in evolution.

## Methods

### Sample collection

Eggs were collected every 24 hours for 6 days on fresh rearing media (95% flour, 5% yeast, by weight) at 30°C from age-matched groups of mated and virgin females from the *GA1* strain of *T. castaneum*. At the time of collection, whole eggs were manually cleaned of excess rearing media and placed into 0.5 ml of Trizol Reagent (Sigma-Aldrich, St. Louis, MO), sheared using a 21 gage needle, and stored at −80°C. Samples from two collection days were combined (days 1 & 4; 2 & 5; 3 & 6) and RNA was isolated using the miRNeasy Mini Kit (Qiagen, Valencia, CA).

### NimbleGen *T. castaneum* whole genome tiled microarray

The microarray consisted of a set of three custom-designed Roche NimbleGen high-density-2 (HD2) whole genome tiling microarray chips, each with 2.1 million isothermal long-oligonucleotide probes (49 – 74 bases range; average 54 bases in length) that sequentially overlap 12 bp, on average. Markov modeled random probes (n=154,000) equivalent in composition to the *T. castaneum* genome sequence were used to set thresholds that measure significant hybridization signals over the background. All experimental probes were designed from unique regions of the genome sequence using the NimbleGen ArrayScribe software and quality assurance tests of the probes were conducted by Center for Genomics and Bioinformatics at Indiana University in-house algorithms [20]. Signal to background ratios were determined by first calling probes that fluoresced at intensities greater than 99% of the random probes’ signal intensities; therefore, only 1% of fluorescing experimental probes above background should be false positives. The arrays reliably produced high signal to background ratios; log_2_ ratios in excess of five were observed for signal over background. We conducted two-color competitive hybridizations that measure differential expression from three replicates, including dye-swaps, each using RNA from independent biological extractions of fertilized and unfertilized eggs.

### RNA sample processing and analysis of data

Beginning with at least 1.0 μg of total RNA, a single round of amplification using MessageAmp™ II aRNA kit (Ambion, Austin, TX) produced more than 100 μg for all samples. Starting with 10 μg of aRNA, double strand cDNA synthesis was carried out using SuperScript Double-Stranded cDNA Synthesis kit (Invitrogen, Carlsbad, CA) using random hexamer primer (Promega, Madison, WI) followed by Dual-Color Labeling Kit (Roche NimbleGen, Madison, WI) using 1 O.D. CY-labeled random nonomer primer per 1 μg double-stranded cDNA in triplicate. Differentially CY-dye labeled treatment and control replicates (48 μg each) were pooled and resuspended with the Hybridization Kit (Roche NimbleGen, Madison, WI). A single dye-swap was included. Hybridization, post-hybridization washing and scanning were done according to the manufacturer’s instructions. Images were acquired using an Axon GenePix 4200A scanner (Molecular Devices, Sunnyvale CA) with GenePix 6.0 software. Data from the images were extracted using NimbleScan 2.4 software (Roche NimbleGen, Madison, WI).

Transcriptionally active regions (TARs) were identified using the approach described in Colbourne, *et al*. [20]. TARs were defined by concatenating overlapping probes showing fluorescence above the 1% false positive rate (FPR). First, replicate arrays were quantile-normalized [28] and to each probe the median value of the replicate probe values was assigned. The fluorescence signal of the random probes, designed to reflect the genome nucleotide composition by Markov modeling, was used to determine a FPR threshold. Probes were considered positive if their fluorescence signal was higher than the 99th percentile of the fluorescence signal of the random probes. TARs were generated using an approach similar to that developed by Kampa, *et al*. [29]. Positive probes were joined into a single TAR if (1) they were adjacent (maxgap=0 bp, no intermittent non-positive probe) and (2) the length was at least 45 bp (minrun=45, mid-point first positive probe to mid-point last positive probe, resulting in at least 3 adjacent positive probes for a TAR). An exon or gene was considered transcribed only if > 80% of its tiled length was expressed.

The data analysis to measure differential expression of genes and of un-annotated TARs was performed using the statistical software package R [30] and Bioconductor [31]. Signal distributions across chips, samples and replicates were adjusted to be equal according to the mean fluorescence of the random probes on each array. All probes (including random probes) were quantile-normalized across replicates. Expression-level scores were assigned for each predicted gene based on the median log_2_ fluorescence over background intensity of probes falling within the exon boundaries. We used the following analysis protocol for estimating differential expression of genes and other genome features from tiled expression data: (1) We created a “tile-expression” table containing normalized log_2_ expression scores for each oligonucleotide probe, with columns for each treatment and replicate, as well as the designated genome location (or address) of each probe; (2) We next created a “tile-gene mapping” table, in the same sorted order as the tile-expression table, which has columns of gene IDs for each exon, intron, tar-region, in rows matching the address of each probe; and, (3) We calculated the per-tile, per-treatment differential expression (DE) levels with LIMMA R package [32]. This balanced-design DE calculation is of the same type that LIMMA is designed to produce.

### Data deposition

The microarray data discussed in this publication have been deposited in NCBI's Gene Expression Omnibus [33] and are accessible through GEO Series accession number GSE38245.

### Expression of maternal *Drosophila melanogaster* orthologs

Lists of *D. melanogaster* maternal genes were obtained from Table S12-S17 of Arbietman, *et al. *[23] Tables two and six of De Renzis, *et al.*[34]. Additional lists of maternal *D. melanogaster* genes were generated with FlyMine v32.0 (http://www.flymine.org, [35]) using the mRNA expression term “maternal” for the Berkeley *Drosophila* Genome Project in situ database [24,25] and Fly-Fish database [26]. BioMart [36] was used to identify *T. castaneum* orthologs to the *D. melanogaster* genes (*Drosophila melanogaster* genes (BDGP 5); ENSEMBL METAZOA 12 (EBI UK) database, Additional file 4). *Drosophila melanogaster* genes without a *T. castaneum* ortholog on the chromosomal assemblies were excluded from further analysis. In each comparison, >90% of all *D. melanogaster* genes with a *T. castaneum* ortholog had only one ortholog. A maternal *D. melanogaster* gene was categorized as having maternal *T. castaneum* orthologs if at least one of the orthologs was expressed in *T. castaneum* unfertilized eggs (Group A). A maternal *D. melanogaster* gene was categorized as having only zygotic *T. castaneum* orthologs if at least one of the orthologs was expressed in *T. castaneum* fertilized eggs, but none in unfertilized eggs (Group B). A maternal *D. melanogaster* gene was categorized as having no expressed orthologs if no *T. castaneum* orthologs were expressed in either fertilized or unfertilized eggs (Group C). The results of the comparisons between the *D. melanogaster* and *T. castaneum* maternal genes were examined for gene ontology germ enrichment using AmiGO (version: 1.8, GO database release 2012-09-08) [37]. Only the Berkeley *Drosophila* Genome Project in situ database [24,25] had a sufficient number of genes and distribution among gene groupings (i.e. Group A, maternally loaded; Group B, fertilized expressed; Group C, not expressed) for the enrichment test to provide enriched terms for all of the comparison groupings. Complete results are provided in the Additional files 1, 2: Table S1, 3 and 4.

### Comparison to previously identified gene groupings in *T. castaneum*

Female-biased expression genes: We analyzed 1661 of the 1821 female-biased expression genes identified in Supplementary Table one of Prince, *et al. *[17] for maternal expression. The remaining 160 genes could not be analyzed because they were located in areas not covered by our microarray (i.e., unassigned contig assemblies). Male-linked genes: Gene datasets from Tables two and three of Parthasarathy, *et al*. [18], Table one of South, *et al*. [19], Supplementary Table one of Prince, *et al*. [17] were compared to the expression results in this study. We substituted TC001980 and TC003552 for TC001930 and TC003522, respectively, in Table one of South, *et al*. [19] based on primer gene identification (A. Dapper, personal communication). Within each dataset, any genes not covered by our microarray were excluded from analysis.

## Abbreviations

DE: Differential expression; FDR: False discovery rate; GO: Gene Ontology; TAR: Transcriptionally active region.

## Competing interests

The authors declare that they have no competing interests.

## Authors' contributions

KMP designed the study, performed sample collection, data analysis, and wrote the paper. JAL performed sample preparation, microarray data collection, and data analysis. JKC assisted in experimental design and writing of the paper. MJW wrote the paper. All authors have read and approved the manuscript.

## Supplementary Material

Additional file 1 Results for individual genes used in the comparisons. Click here for file

Additional file 2 **Table S1.** Previously identified early *T. castaneum* developmental gene expression results. Click here for file

Additional file 3 Complete results of the gene ontology term enrichment analysis. Click here for file

Additional file 4 **Orthologous *T. castaneum *gene ID numbers, *T. castaneum *gene names/aliases, *D. melanogaster *FlyBase Gene ID numbers, and *D. melanogaster *gene names or annotation symbols.** Click here for file
