# Supplementary material for: Identification of maternally-loaded RNA transcripts in unfertilized eggs of Tribolium castaneum
Source: BMC Genomics. 2012 Nov 27;13:671. doi: 10.1186/1471-2164-13-671 (PMC3536564; doi:10.1186/1471-2164-13-671)
Supplement: Additional file 2 — Table S1. Previously identified early T. castaneum developmental gene expression results. [file 1471-2164-13-671-S2.pdf]

Table S1. Previously identified early *T. castaneum* developmental gene expression results.

Gene (Symbol)	Gene ID	Previous Reported Expression Stage	Reference	Unfertilized Egg Microarray Expression	Fertilized Egg Microarray Expression
hunchback (*hb*)	TC013553	maternal	[1]	Yes	Yes
caudal (*cad*)	TC007576	maternal	[2]	Yes	Yes
dorsal (*Dl*)	TC007697	maternal	[3]	Yes	Yes
zerknullt (*zen*)	TC000921	maternal	[4]	Yes	Yes
torso (*tor*)	TC009906	maternal	[5]	Yes	Yes
orthodenticle-1 (*otd1*)	TC003354	maternal	[6]	Yes	Yes
eagle (*Eg*)	TC003409	maternal	[7]	Yes	Yes
pangolin (*Pan*)	TC003728	maternal	[7]	Yes	Yes
knirps (*Kni*)	TC003413	blastoderm	[8]	No	Yes
maxillopedia (*mxp*)	TC000925	late blastoderm	[9]	No	Yes

References:

1. Wolff C, Sommer R, Schroder R, Glaser G, Tautz D: **Conserved and divergent expression aspects of the Drosophila segmentation gene hunchback in the short germ band embryo of the flour beetle Tribolium.** *Development* 1995, **121:**4227-4236.

2. Schulz C, Schroder R, Hausdorf B, Wolff C, Tautz D: **A caudal homologue in the short germ band beetle Tribolium shows similarities to both, the Drosophila and the vertebrate caudal expression patterns.** *Dev Genes Evol* 1998, **208:**283-289.

3. Chen G, Handel K, Roth S: **The maternal NF-kappaB/dorsal gradient of Tribolium castaneum: dynamics of early dorsoventral patterning in a short-germ beetle.** *Development* 2000, **127:**5145-5156.

4. van der Zee M, Berns N, Roth S: **Distinct functions of the Tribolium zerknullt genes in serosa specification and dorsal closure.** *Curr Biol* 2005, **15:**624-636.

5. Schoppmeier M, Schroder R: **Maternal torso signaling controls body axis elongation in a short germ insect.** *Curr Biol* 2005, **15:**2131-2136.

6. Schroder R: **The genes orthodenticle and hunchback substitute for bicoid in the beetle Tribolium.** *Nature* 2003, **422:**621-625.

7. Bucher G, Farzana L, Brown SJ, Klingler M: **Anterior localization of maternal mRNAs in a short germ insect lacking bicoid.** *Evol Dev* 2005, **7:**142-149.

8. Cerny AC, Grossmann D, Bucher G, Klingler M: **The Tribolium ortholog of knirps and knirps-related is crucial for head segmentation but plays a minor role during abdominal patterning.** *Dev Biol* 2008, **321:**284-294.

9. Shippy TD, Guo J, Brown SJ, Beeman RW, Denell RE: **Analysis of maxillopedia expression pattern and larval cuticular phenotype in wild-type and mutant tribolium.** *Genetics* 2000, **155:**721-731.
