# Supplementary material for: Identification of maternally-loaded RNA transcripts in unfertilized eggs of Tribolium castaneum
Source: BMC Genomics. 2012 Nov 27;13:671. doi: 10.1186/1471-2164-13-671 (PMC3536564; doi:10.1186/1471-2164-13-671)
Supplement: Additional file 3 — Complete results of the gene ontology term enrichment analysis. [file 1471-2164-13-671-S3.pdf]

GO term enrichment of the fly-beetle comparisons

Berkeley *Drosophila* Genome Project in situ database

[1, 2]

Fertilized only:

Molecular Functions

GO Term	Aspect	P-value	Sample frequency	Background frequency
GO:0003700 sequence-specific DNA binding transcription factor activity	F	5.44e-04	35/184 (19.0%)	290/3718 (7.8%)
GO:0001071 nucleic acid binding transcription factor activity	F	5.44e-04	35/184 (19.0%)	290/3718 (7.8%)
GO:0005214 structural constituent of chitin-based cuticle	F	4.36e-03	11/184 (6.0%)	41/3718 (1.1%)
GO:0042302 structural constituent of cuticle	F	7.28e-03	11/184 (6.0%)	43/3718 (1.2%)

Maternal Loaded:

Biological Prosses:

GO Term	Aspect	P-value	Sample frequency	Background frequency
GO:0009987 cellular process	P	6.59e-16	2309/3235 (71.4%)	2552/3718 (68.6%)
GO:0044237 cellular metabolic process	P	6.62e-15	1493/3235 (46.2%)	1616/3718 (43.5%)
GO:0044267 cellular protein metabolic process	P	9.59e-13	567/3235 (17.5%)	589/3718 (15.8%)
GO:0006412 translation	P	5.87e-10	207/3235 (6.4%)	207/3718 (5.6%)
GO:0022402 cell cycle process	P	6.84e-10	284/3235 (8.8%)	288/3718 (7.7%)
GO:0007049 cell cycle	P	1.37e-09	324/3235 (10.0%)	331/3718 (8.9%)
GO:0044260 cellular macromolecule metabolic process	P	7.08e-09	1147/3235 (35.5%)	1243/3718 (33.4%)
GO:0022403 cell cycle phase	P	2.24e-	242/3235	245/3718 (6.6%)

GO Term	Aspect	P-value	Sample frequency	Background frequency
		08	(7.5%)	
GO:0006996 organelle organization	P	2.42e-08	480/3235 (14.8%)	502/3718 (13.5%)
GO:0000279 M phase	P	2.31e-06	191/3235 (5.9%)	193/3718 (5.2%)
GO:0006396 RNA processing	P	1.18e-05	179/3235 (5.5%)	181/3718 (4.9%)
GO:0000278 mitotic cell cycle	P	1.92e-05	235/3235 (7.3%)	241/3718 (6.5%)
GO:0071841 cellular component organization or biogenesis at cellular level	P	2.23e-04	725/3235 (22.4%)	784/3718 (21.1%)
GO:0008152 metabolic process	P	5.57e-04	1870/3235 (57.8%)	2088/3718 (56.2%)
GO:0006974 response to DNA damage stimulus	P	8.15e-04	110/3235 (3.4%)	110/3718 (3.0%)
GO:0033036 macromolecule localization	P	9.50e-04	251/3235 (7.8%)	261/3718 (7.0%)
GO:0071842 cellular component organization at cellular level	P	1.80e-03	698/3235 (21.6%)	757/3718 (20.4%)
GO:0016071 mRNA metabolic process	P	2.14e-03	140/3235 (4.3%)	142/3718 (3.8%)
GO:0000226 microtubule cytoskeleton organization	P	2.37e-03	182/3235 (5.6%)	187/3718 (5.0%)
GO:0007051 spindle organization	P	3.79e-03	119/3235 (3.7%)	120/3718 (3.2%)
GO:0006397 mRNA processing	P	5.34e-03	133/3235 (4.1%)	135/3718 (3.6%)
GO:0033554 cellular response to stress	P	5.44e-03	175/3235 (5.4%)	180/3718 (4.8%)
GO:0006259 DNA metabolic process	P	7.47e-03	114/3235 (3.5%)	115/3718 (3.1%)
GO:0051641 cellular localization	P	7.82e-03	264/3235 (8.2%)	277/3718 (7.5%)

Cellular Component:

GO Term	Aspect	P-value	Sample frequency	Background frequency
GO:0044446 intracellular organelle part	C	2.30e-24	849/3235 (26.2%)	878/3718 (23.6%)

GO Term	Aspect	P-value	Sample frequency	Background frequency
GO:0044422 organelle part	C	1.88e-23	856/3235 (26.5%)	887/3718 (23.9%)
GO:0005622 intracellular	C	1.99e-17	1774/3235 (54.8%)	1929/3718 (51.9%)
GO:0044424 intracellular part	C	4.17e-17	1662/3235 (51.4%)	1802/3718 (48.5%)
GO:0005737 cytoplasm	C	1.37e-14	968/3235 (29.9%)	1027/3718 (27.6%)
GO:0032991 macromolecular complex	C	3.91e-13	847/3235 (26.2%)	896/3718 (24.1%)
GO:0044428 nuclear part	C	4.42e-13	369/3235 (11.4%)	375/3718 (10.1%)
GO:0044444 cytoplasmic part	C	7.10e-12	726/3235 (22.4%)	765/3718 (20.6%)
GO:0043226 organelle	C	2.27e-11	1362/3235 (42.1%)	1478/3718 (39.8%)
GO:0043229 intracellular organelle	C	2.50e-11	1361/3235 (42.1%)	1477/3718 (39.7%)
GO:0031974 membrane-enclosed lumen	C	1.50e-10	311/3235 (9.6%)	316/3718 (8.5%)
GO:0070013 intracellular organelle lumen	C	2.95e-10	306/3235 (9.5%)	311/3718 (8.4%)
GO:0043233 organelle lumen	C	2.95e-10	306/3235 (9.5%)	311/3718 (8.4%)
GO:0030529 ribonucleoprotein complex	C	6.35e-09	214/3235 (6.6%)	215/3718 (5.8%)
GO:0043227 membrane-bounded organelle	C	1.29e-08	1156/3235 (35.7%)	1254/3718 (33.7%)
GO:0043231 intracellular membrane-bounded organelle	C	1.41e-08	1155/3235 (35.7%)	1253/3718 (33.7%)
GO:0005623 cell	C	1.51e-07	1910/3235 (59.0%)	2118/3718 (57.0%)
GO:0044464 cell part	C	1.51e-07	1910/3235 (59.0%)	2118/3718 (57.0%)
GO:0031981 nuclear lumen	C	1.24e-06	228/3235 (7.0%)	232/3718 (6.2%)
GO:0043234 protein complex	C	4.57e-06	650/3235 (20.1%)	695/3718 (18.7%)
GO:0043228 non-membrane-bounded organelle	C	1.46e-	460/3235	486/3718 (13.1%)

GO Term	Aspect	P-value	Sample frequency	Background frequency
		05	(14.2%)	
GO:0043232 intracellular non-membrane-bounded organelle	C	1.46e-05	460/3235 (14.2%)	486/3718 (13.1%)
GO:0005829 cytosol	C	1.45e-04	122/3235 (3.8%)	122/3718 (3.3%)
GO:0031090 organelle membrane	C	3.38e-04	154/3235 (4.8%)	156/3718 (4.2%)
GO:0044429 mitochondrial part	C	9.03e-04	162/3235 (5.0%)	165/3718 (4.4%)
GO:0005654 nucleoplasm	C	2.25e-03	169/3235 (5.2%)	173/3718 (4.7%)
GO:0005739 mitochondrion	C	3.71e-03	227/3235 (7.0%)	236/3718 (6.3%)

Molecular Function:

GO Term	Aspect	P-value	Sample frequency	Background frequency
GO:0036094 small molecule binding	F	5.17e-08	525/3235 (16.2%)	552/3718 (14.8%)
GO:1901265 nucleoside phosphate binding	F	1.24e-07	486/3235 (15.0%)	510/3718 (13.7%)
GO:0000166 nucleotide binding	F	1.24e-07	486/3235 (15.0%)	510/3718 (13.7%)
GO:0097159 organic cyclic compound binding	F	1.24e-07	486/3235 (15.0%)	510/3718 (13.7%)
GO:0003723 RNA binding	F	1.42e-05	194/3235 (6.0%)	197/3718 (5.3%)
GO:0035639 purine ribonucleoside triphosphate binding	F	5.19e-04	333/3235 (10.3%)	350/3718 (9.4%)
GO:0032555 purine ribonucleotide binding	F	5.19e-04	333/3235 (10.3%)	350/3718 (9.4%)
GO:0032553 ribonucleotide binding	F	5.19e-04	333/3235 (10.3%)	350/3718 (9.4%)
GO:0017076 purine nucleotide binding	F	1.07e-03	336/3235 (10.4%)	354/3718 (9.5%)
GO:0016740 transferase activity	F	2.35e-03	387/3235 (12.0%)	411/3718 (11.1%)

Not Expressed:

Biological Processes:

GO Term	Aspect	P-value	Sample frequency	Background frequency
GO:0006508 proteolysis	P	1.84e-04	41/299 (13.7%)	218/3718 (5.9%)
GO:0007600 sensory perception	P	3.53e-03	16/299 (5.4%)	53/3718 (1.4%)

Cellular Components:

GO Term	Aspect	P-value	Sample frequency	Background frequency
GO:0005887 integral to plasma membrane	C	5.42e-06	23/299 (7.7%)	72/3718 (1.9%)
GO:0031226 intrinsic to plasma membrane	C	9.89e-06	23/299 (7.7%)	74/3718 (2.0%)
GO:0016021 integral to membrane	C	5.40e-03	54/299 (18.1%)	364/3718 (9.8%)
GO:0031224 intrinsic to membrane	C	9.04e-03	54/299 (18.1%)	370/3718 (10.0%)

Molecular Functions:

GO Term	Aspect	P-value	Sample frequency	Background frequency
GO:0004175 endopeptidase activity	F	6.26e-10	37/299 (12.4%)	124/3718 (3.3%)
GO:0004252 serine-type endopeptidase activity	F	5.99e-09	21/299 (7.0%)	45/3718 (1.2%)
GO:0070011 peptidase activity, acting on L-amino acid peptides	F	8.20e-08	41/299 (13.7%)	171/3718 (4.6%)
GO:0008236 serine-type peptidase activity	F	4.22e-07	21/299 (7.0%)	54/3718 (1.5%)
GO:0017171 serine hydrolase activity	F	6.33e-07	21/299 (7.0%)	55/3718 (1.5%)
GO:0008233 peptidase activity	F	7.99e-07	41/299 (13.7%)	183/3718 (4.9%)
GO:0022836 gated channel activity	F	4.47e-06	15/299 (5.0%)	31/3718 (0.8%)
GO:0004222metalloendopeptidase activity	F	2.00e-04	14/299 (4.7%)	34/3718 (0.9%)

GO Term	Aspect	P-value	Sample frequency	Background frequency
GO:0005216 ion channel activity	F	3.06e-04	15/299 (5.0%)	40/3718 (1.1%)
GO:0060089 molecular transducer activity	F	4.18e-04	27/299 (9.0%)	116/3718 (3.1%)
GO:0004871 signal transducer activity	F	4.18e-04	27/299 (9.0%)	116/3718 (3.1%)
GO:0004872 receptor activity	F	5.07e-04	25/299 (8.4%)	103/3718 (2.8%)
GO:0022838 substrate-specific channel activity	F	9.22e-04	15/299 (5.0%)	43/3718 (1.2%)
GO:0042302 structural constituent of cuticle	F	9.22e-04	15/299 (5.0%)	43/3718 (1.2%)
GO:0004888 transmembrane signaling receptor activity	F	1.11e-03	19/299 (6.4%)	67/3718 (1.8%)
GO:0022834 ligand-gated channel activity	F	1.56e-03	10/299 (3.3%)	20/3718 (0.5%)
GO:0015276 ligand-gated ion channel activity	F	1.56e-03	10/299 (3.3%)	20/3718 (0.5%)
GO:0038023 signaling receptor activity	F	2.28e-03	21/299 (7.0%)	83/3718 (2.2%)
GO:0005214 structural constituent of chitin-based cuticle	F	3.01e-03	14/299 (4.7%)	41/3718 (1.1%)
GO:0022803 passive transmembrane transporter activity	F	3.40e-03	15/299 (5.0%)	47/3718 (1.3%)
GO:0015267 channel activity	F	3.40e-03	15/299 (5.0%)	47/3718 (1.3%)
GO:0008237 metallopeptidase activity	F	3.47e-03	17/299 (5.7%)	59/3718 (1.6%)

Fly-Fish database

[3]

Fertilized only:

No enriched terms

Maternal Loaded:

Cellular Components:

GO Term	Aspect	P-value	Sample frequency	Background frequency
GO:0005622 intracellular	C	5.28e-05	669/1163 (57.5%)	703/1276 (55.1%)
GO:0044446 intracellular organelle part	C	5.95e-05	340/1163 (29.2%)	348/1276 (27.3%)
GO:0044424 intracellular part	C	1.51e-04	626/1163 (53.8%)	657/1276 (51.5%)
GO:0044422 organelle part	C	2.45e-04	341/1163 (29.3%)	350/1276 (27.4%)
GO:0044428 nuclear part	C	6.60e-03	160/1163 (13.8%)	161/1276 (12.6%)
GO:0005737 cytoplasm	C	6.85e-03	362/1163 (31.1%)	375/1276 (29.4%)

Not expressed:

No enriched Terms

De Renzis, *et al.*

De Renzis, *et al.* [4]

Fertilized only: no enriched terms
Maternal loaded: no enriched terms
Not expressed: no enriched terms

Arbeitman, *et al.*

Arbeitman, *et al.* [5]

Fertilized only: no enriched terms
Maternal loaded: no enriched terms
Not expressed: no enriched terms

References:

1. Tomancak P, Beaton A, Weiszmann R, Kwan E, Shu S, Lewis SE, Richards S, Ashburner M, Hartenstein V, Celniker SE, Rubin GM: **Systematic determination of patterns of gene expression during Drosophila embryogenesis.** *Genome Biology* 2002, **3:**RESEARCH0088.

2. Tomancak P, Berman BP, Beaton A, Weiszmann R, Kwan E, Hartenstein V, Celniker SE, Rubin GM: **Global analysis of patterns of gene expression during Drosophila embryogenesis.** *Genome Biology* 2007, **8**.

3. Lecuyer E, Yoshida H, Parthasarathy N, Alm C, Babak T, Cerovina T, Hughes TR, Tomancak P, Krause HM: **Global analysis of mRNA localization reveals a prominent role in organizing cellular architecture and function.** *Cell* 2007, **131:**174-187.

4. De Renzis S, Elemento O, Tavazoie S, Wieschaus EF: **Unmasking activation of the zygotic genome using chromosomal deletions in the Drosophila embryo.** *Plos Biology* 2007, **5:**1036-1051.

5. Arbeitman MN, Furlong EEM, Imam F, Johnson E, Null BH, Baker BS, Krasnow MA, Scott MP, Davis RW, White KP: **Gene expression during the life cycle of Drosophila melanogaster.** *Science* 2002, **297:**2270-2275.
